# Capturing cell morphology dynamics with high temporal resolution using single-shot quantitative phase gradient imaging

**DOI:** 10.1117/1.JBO.29.S2.S22712

**Published:** 2024-07-16

**Authors:** Sun Woong Hur, Minsung Kwon, Revathi Manoharaan, Melika Haji Mohammadi, Ashok Zachariah Samuel, Michael P. Mulligan, Paul J. Hergenrother, Rohit Bhargava

**Affiliations:** a University of Illinois at Urbana-Champaign, Department of Bioengineering, Urbana, Illinois, United States; b University of Illinois at Urbana-Champaign, Beckman Institute for Advanced Science and Technology, Urbana, Illinois, United States; c University of Illinois at Urbana-Champaign, Department of Chemistry, Urbana, Illinois, United States; d University of Illinois Urbana-Champaign, Carl R. Woese Institute for Genomic Biology, Urbana, Illinois, United States; e University of Illinois Urbana-Champaign, Cancer Center at Illinois, Urbana, Illinois, United States; f University of Illinois Urbana-Champaign, Department of Chemical and Biomolecular Engineering, Electrical and Computer Engineering, Mechanical Science and Engineering and Chemistry, Urbana, Illinois, United States

**Keywords:** label-free imaging, quantitative phase imaging, cell death, morphology, single-shot imaging

## Abstract

**Significance:**

Label-free quantitative phase imaging can potentially measure cellular dynamics with minimal perturbation, motivating efforts to develop faster and more sensitive instrumentation. We characterize fast, single-shot quantitative phase gradient microscopy (ss-QPGM) that simultaneously acquires multiple polarization components required to reconstruct phase images. We integrate a computationally efficient least squares algorithm to provide real-time, video-rate imaging (up to 75  frames/s). The developed instrument was used to observe changes in cellular morphology and correlate these to molecular measures commonly obtained by staining.

**Aim:**

We aim to characterize a fast approach to ss-QPGM and record morphological changes in single-cell phase images. We also correlate these with biochemical changes indicating cell death using concurrently acquired fluorescence images.

**Approach:**

Here, we examine nutrient deprivation and anticancer drug-induced cell death in two different breast cell lines, *viz.*, M2 and MCF7. Our approach involves in-line measurements of ss-QPGM and fluorescence imaging of the cells biochemically labeled for viability.

**Results:**

We validate the accuracy of the phase measurement using a USAF1951 pattern phase target. The ss-QPGM system resolves 912.3  lp/mm, and our analysis scheme accurately retrieves the phase with a high correlation coefficient (∼0.99), as measured by calibrated sample thicknesses. Analyzing the contrast in phase, we estimate the spatial resolution achievable to be 0.55  μm for this microscope. ss-QPGM time-lapse live-cell imaging reveals multiple intracellular and morphological changes during biochemically induced cell death. Inferences from co-registered images of quantitative phase and fluorescence suggest the possibility of necrosis, which agrees with previous findings.

**Conclusions:**

Label-free ss-QPGM with high-temporal resolution and high spatial fidelity is demonstrated. Its application for monitoring dynamic changes in live cells offers promising prospects.

## 1 Introduction

Quantitative phase imaging (QPI) is a promising method for real-time, label-free, and high-contrast imaging^1^ that typically measures the phase delay of light transmitted through largely transparent and weakly scattering samples.^2^^,^^3^ Several interferometric instrumental techniques have been developed for QPI, such as phase-shifting interferometry,^4^ digital holography,^5^ Hilbert phase microscopy,^6^ and low-coherence interferometry.^7^ These sensitive measurement techniques require high-precision optical alignment, and the fidelity of recorded data may suffer from artifacts arising from fluctuations in phase, polarization, and source coherence. Non-interferometric QPI methods, such as differential phase-contrast microscopy,^8^ Fourier ptychography,^9^ and transport of the intensity estimations,^10^ have been proposed to overcome the limitations of interferometric recording. However, these measurement schemes require either sequential intensity measurements or extensive computational effort to fully reconstruct the phase information from the nonlinear relationship between phase and intensity. Among recent advancements in obtaining rapid and high-quality QPI measurements is gradient light interference microscopy,^11^ which measures four phase-shifted light intensities using a spatial light modulator and applies a Hilbert transform to recover the phase. An alternative approach that achieved single-shot QPI utilizing a quarter-wave plate and a polarization camera has been demonstrated.^12^ Since the integration of quantitative phase gradients can induce image artifacts, the authors employed the Alternative Direction Method of Multipliers-based algorithm for phase recovery in this work; however, this processing requires extensive computing resources and time. Efforts to achieve real-time QPI images using light-emitting diode (LED)-based color-multiplexing,^13^^,^^14^ polarization multiplexing,^15^ or meta-surfaces^16^ have also been reported. Another approach may be to use deep learning methods^17^ to estimate phase from single-shot measurements, but these methods involve extensive training and may be limited to the sample types used during the training.

In this study, we have implemented a microscope for a single-shot quantitative phase gradient microscopy (ss-QPGM) by modifying a conventional differential interference contrast (DIC) microscope. This involved removing the analyzer and adding an imaging module that includes a quarter-wave plate and a complementary metal–oxide–semiconductor (CMOS) sensor with an integrated polarization filter array. We obtained quantitative phase gradients from a single intensity measurement and computed quantitative phase images using the least squares algorithm capable of real-time image reconstruction. We characterized ss-QPGM with a standard phase target and demonstrated its utility in cell biology by observing live human cells. The image contrast in ss-QPGM originates from the phase delay induced by variations in the refractive index and sample thickness differences at different locations within biological cells.^18^^,^^19^ This approach effectively reveals morphological features of cells, which has been widely validated to reveal cellular dynamics, including estimating cell mass and growth-rate changes,^18^^–^^20^ cell death dynamics,^21^ motility and invasion,^22^ and progression through the cell cycle.^23^ The label-free and fast imaging capability can be potentially useful in the screening of therapeutics using morphological and phenotypic alterations at the single-cell level.^18^ Here, we investigate morphological dynamics during cell death induced by nutrient deprivation as well as a novel anticancer drug candidate (ErSO) using ss-QPGM. During programmed (e.g., apoptosis) and non-programmed (e.g., necrosis) cell death, cells transition through multiple stages of morphological and biochemical alterations. We use time-lapse ss-QPGM images to record the process of cell death in real time, focusing on known morphologic features, including enhanced intracellular granularity, blebbing, and membrane rupture.

## 2 Methods

### 2.1 Experimental Setup

**Fig. 1:**
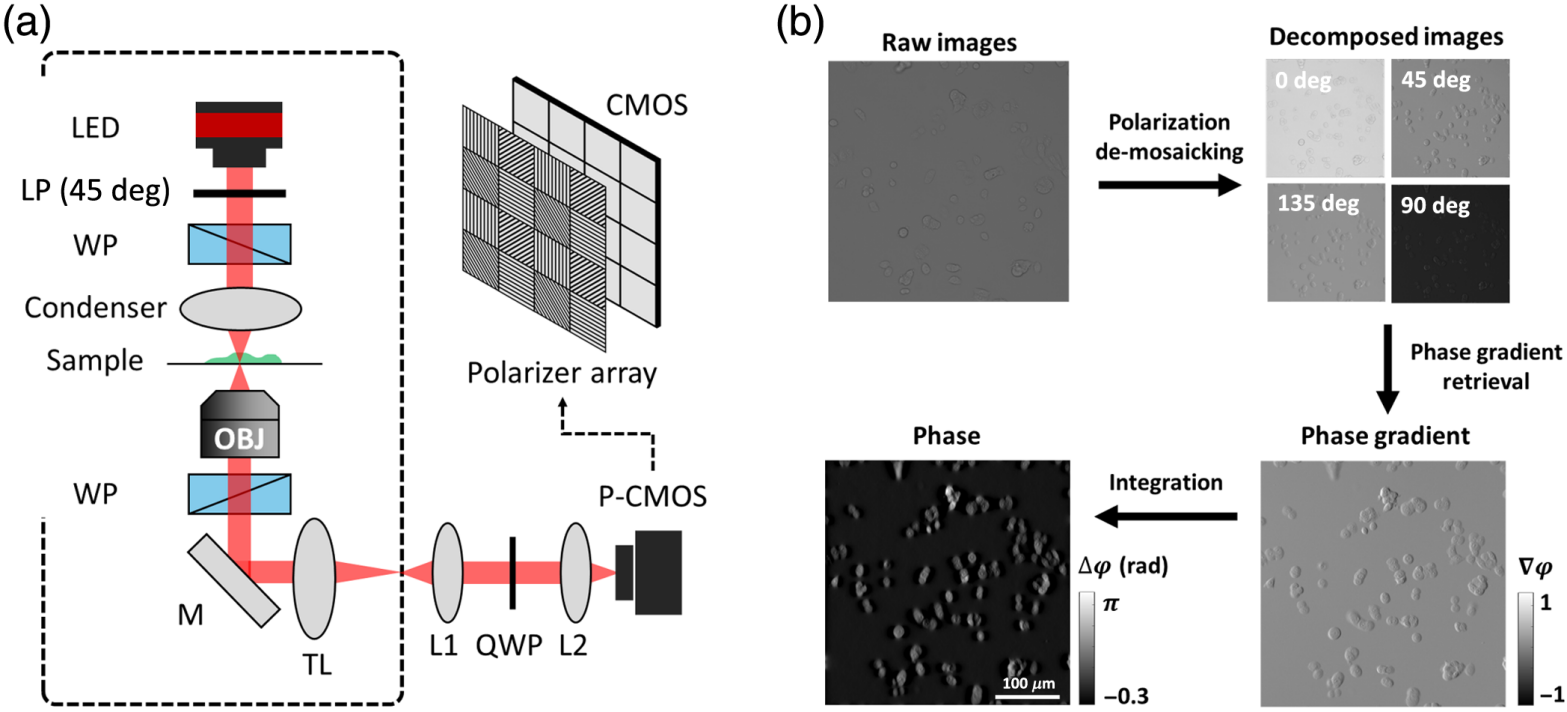
Schematic of ss-QPGM instrumentation and data processing workflow. (a) The instrument consists of a typical DIC microscope, as shown by the dashed black line, with a ss-QPI module added. The most critical addition is the use of a polarization filter array positioned in front of the CMOS sensor array. P-CMOS, polarization-sensitive CMOS camera; LP, linear polarizer; WP, Wollaston prism; TL, tube lens; M, mirror; L, lens; QWP, quarter-wave plate. (b) The workflow to obtain quantitative phase data consists of four major steps. First, the acquired data are de-mosaicked into four individual images. Second, spatial frequency-preserving interpolation is used to obtain full field-of-view images for each polarization. Third, a phase gradient image is computed from the four images; fourth, the phase image is estimated through integration.

The optical system for ss-QPGM was built as a functional extension of a DIC microscope,^11^^,^^12^ with the configuration shown in Fig. 1(a). A high-power LED (SOLIS-660C, Thorlabs, Newton, New Jersey, United States), with a center wavelength of 670 nm and a spectral bandwidth of 21 nm, was used as the light source. The LED input was split into two orthogonal linearly polarized beams using a linear polarizer and a Wollaston prism. The two beams, separated by a distance smaller than the system’s diffraction limit, were collected by a 20×/0.5NA objective lens (Zeiss, Oberkochen, Baden-Württemberg, Germany) after traversing an object. A second Wollaston prism was used to combine the orthogonally polarized transmitted light at the back focal plane of the objective lens. The combined beam was focused onto the intermediate image plane and projected by the telescope consisting of L1 and L2 lenses (AC508-080-A-ML, Thorlabs Inc.) onto the detector. A quarter-wave plate (Edmund Optics Inc., Barrington, New Jersey, United States) was placed at the Fourier plane of the telescope to convert the linearly polarized beams into the corresponding left and right circular polarized light, which are detected using a polarization CMOS sensor (BFS-U3-51S5P-C, FLIR, Wilsonville, Oregon, United States; 2448×2048; pixel size of 3.45  μm). For cell imaging, we acquired images at a rate of 15  frames/s to manage data volumes; however, the optical setup can capture up to 75  frames/s. The ss-QPGM optical system is implemented on an inverted microscope (Observer Z1, Zeiss), which also permits in-line fluorescence imaging. By switching the optical path to two imaging ports of the microscope, we alternately performed ss-QPGM and fluorescence imaging in the same field of view. The wide-field fluorescence imaging was conducted for cell viability assay with the use of the monochrome camera (Orca-Flash 4.0, Hamamatsu, Japan) at a different imaging port. From the rear side of the microscope, a typical metal halide lamp (X-cite 120Q, Excelitas, Pittsburgh, Pennsylvania, United States) was guided with a light guide and filtered by the two filter tubes. Each cube consists of an excitation filter, a dichroic filter, and an emission filter. Cube 1, which includes a BP475/35 excitation filter, a 499 dichroic filter, and a BP530/43 emission filter, was used for live green imaging. Cube 2, featuring a BP540/25 excitation filter, a 565 dichroic mirror, and a BP605/55 emission filter, was used for dead red imaging.

### 2.2 Data Processing

The data processing workflow is shown in Fig. 1(b). The raw image is mosaicked by a polarization filter array and contains four polarization states (0, 45, 90, and 135 deg) for every 4 pixels. We reconstitute the full 2048×2048 image for each polarization channel by applying Newton’s polynomial interpolation model^24^ while preserving high- and low-frequency feature information. Intensities from these polarization channels are then utilized to compute quantitative phase gradient images. The phase gradient values are integrated to estimate phase images. The basis of our processing is the forward model of image formation in ss-QPGM. The light field at the intermediate image plane is defined^25^^,^^26^ as

U(r)=o^·A(r−Δr)eiϕ(r−Δr)+e^·A(r+Δr)eiϕ(r+Δr), (1)

where r indicates two-dimensional spatial coordinates; $\hat{o}$ and $\hat{e}$ are vectorized expressions for ordinary and extraordinary rays, respectively; A(r) and ϕ(r) are the amplitude and the phase of light, respectively; and 2Δr denotes spatial shear vector induced by Wollaston prisms in DIC. The field $E_{\text{out},\theta }$ at the image plane can be represented by a Jones matrix

$$E_{\text{out},\theta }=L_{\theta }Q_{45}U(r),$$ (2)

where $L_{\theta }=(\begin{array}{cc} \cos^{2}\;\theta & \cos\;\theta \cdot \sin\;\theta \\ \cos\;\theta \cdot \sin\;\theta & \sin^{2}\;\theta \end{array})$ is a linear polarizer matrix with axis angle θ and $Q_{45}=e^{\frac{-i\pi }{4}}(\begin{array}{cc} 1+i & 1-i\\ 1-i & 1+i \end{array})$ is the Quarter-wave plate with angle of 45 deg with respect to fast axis.^27^ The intensity of each polarization channel is given by, $I_{\theta }={\left|E_{\text{out},\theta }\right|}^{2}$. The quantitative phase gradient image is computed with acquired polarization channels as

$$\phi (r+\Delta r)-\phi (r-\Delta r)=\tan^{-1}(\frac{I_{45}-I_{135}}{I_{0}-I_{90}}).$$ (3)

Among many different ways of computing integration, we adopted deconvolution using a defined transfer function of the gradient operator, ${\tilde{H}}_{r}(u)$, which is 2j sin(2πu·Δr)=F{δ(r+Δr)−δ(r−Δr)}, where u indicates the spatial frequency coordinates and F is the Fourier transform

$$F\{\phi (r+\Delta r)-\phi (r-\Delta r)\}={\tilde{H}}_{r}(u)\cdot \tilde{\phi }(u),$$ (4)

where $\tilde{\phi }$ is the Fourier transform of the phase. Since the transfer function has many zero values, direct deconvolution cannot produce a correct estimation and amplifies the noise at corresponding frequencies. Thus, we formulated the least-squares approach to integrate the phase gradients using Tikhonov regularization, commonly used for conventional QPI^8^^,^^28^^,^^29^

$$\underset{\phi }{\min}\;{\left\|\nabla_{r}\overset{˜}{\phi }(u)-[{\overset{˜}{H}}_{r}(u)\cdot \overset{˜}{\phi }(u)]\right\|}^{2}+\alpha {\left|\overset{˜}{\phi }(u)\right|}^{2},$$ (5)

where α is the regularization parameter; $\nabla_{r}\tilde{\phi }(u)=F\{\phi (r+\Delta r)-\phi (r-\Delta r)\}$ is the Fourier transform of phase gradient. The quantitative phase image is obtained by

$$\phi (r)=F^{-1}\{\frac{{\overset{˜}{H}}_{r}^{*}(u)\cdot \nabla_{r}\overset{˜}{\phi }(u)}{|{\overset{˜}{H}}_{r}(u){|}^{2}+\alpha }\},$$ (6)

where ${\tilde{H}}_{r}^{*}(u)$ denotes the complex conjugate of the transfer function of the gradient operator. We empirically estimated the optimal value of $\alpha =3\times 10^{-4}$ by performing phase imaging with a calibrated phase target; this value was used for reconstructing QPI images of the biological samples.

### 2.3 Cell Culture

Two cell lines, namely, MCF10AT1k.cl2 (M2)^30^ and MCF7,^31^ representing premalignant and low-metastatic breast cancer, respectively, were chosen for the present study. M2 cells were maintained in $75\;{\mathrm{cm}}^{2}$ flasks in DMEM/F12 medium (Gibco 11320033, Grand Island, New York, United States) supplemented with 5% horse serum (Gibco 16050114), 20  ng/mL epidermal growth factor (Sigma 5036, Marietta, Georgia, United States), 0.5  mg/mL hydrocortisone (Sigma H0888), 100  ng/mL cholera toxin (Sigma C8052), 10  μg/mL insulin (Sigma I1882), and 1% penicillin–streptomycin (Fisher I7602E, Hampton, New Hampshire, United States). MCF7 cells were maintained in $75\;{\mathrm{cm}}^{2}$ flasks in RPMI 1640 medium (Gibco 11879020) supplemented with 5% fetal bovine serum (Gibco A31605-01) and 1% penicillin–streptomycin (Fisher I7602E). The cells were cultured in a 5% ${\mathrm{CO}}_{2}$ incubator at 37°C and passaged at 70% confluency. The passage number was subjected to less than nine in the experiments to avoid any genetic drift. Cells were seeded in glass-bottom six-well plates (Cellvis NC0454735, Mountain View, California, United States) at a density of $10^{4}\text{ cells}/\mathrm{mL}$ and cultured overnight to reach the confluency needed for the experiment. The cell seeding density was carefully controlled such that the cells were deposited as a monolayer.

M2 cells were subjected to nutrient-deprived culture conditions by incubating them in phosphate-buffered saline (PBS) for 3 h to understand cell death events. PBS has a balanced saline concentration to prevent osmotic shock and buffering agents to stabilize pH, but it lacks essential nutrients and growth factors. PBS is occasionally used for securely maintaining cell quiescence during passages in cell culture. MCF7 cells were treated with 100  μM of ErSO for the measurements. LIVE/DEAD™ Viability/Cytotoxicity Kit for mammalian cells (Invitrogen L3224, Waltham, Massachusetts, United States; contains 2  μM calcein AM and 4  μM ethidium bromide) was used for performing cell viability assay with fluorescence imaging. In this procedure, live and dead cells get labeled with calcein (green) and ethidium bromide (red), respectively.

## 3 Results

### 3.1 Performance Validation Using a Calibrated Phase Target

**Fig. 2:**
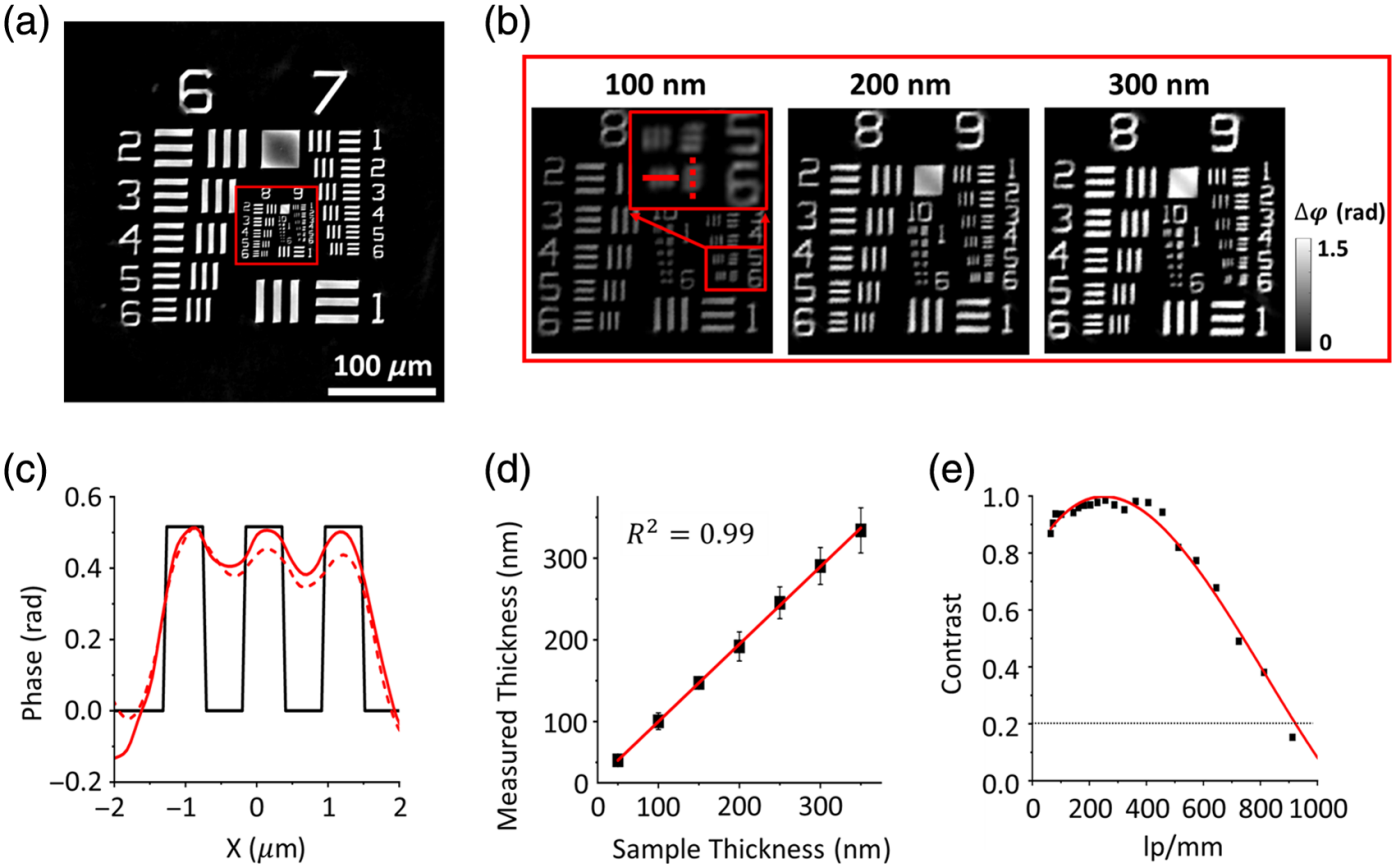
Quantitative measures of performance for ss-QPGM. (a) Reconstructed quantitative phase image of the calibration phase target. (b) Magnified images of the location marked with the red solid rectangles in panel (a). (c) Line profiles of the phase along the red solid and dashed lines in magnified boxes in panel (b). The red solid line and dashed line are profiles along the x-axis and y-axis, respectively. (d) The calculated thickness of the phase targets with seven different thicknesses by ss-QPGM. (e) MTF curves of ss-QPGM.

We validated the performance of ss-QPGM using a phase-calibrated USAF1951 microscopy target (Benchmark Technologies, Lynnfield, Massachusetts, United States) by examining various quantitative metrics, including spatial resolution, spatial fidelity, and modulation transfer function. To assess the imaging performance, we focused on vertical and horizontal features at discrete spatial frequencies within the standard sample. First, we used this target to demonstrate the accuracy of phase retrieval. Figure 2(a) shows a quantitative phase image of the target obtained from our system, as recovered by applying the phase retrieval algorithm [Eqs. (4) and (5)]. To illustrate the feasibility of phase images with different thicknesses of samples, we examined magnified regions of the image, focusing on groups 9 and 10 [Fig. 2(b)]. Line profiles of the values from group 9, element 6 (0.55  μm line width) are shown in Fig. 2(c). The line profiles show good correspondence between the reconstructed phase (red solid line: x-axis and red dashed line: y-axis) and the designed feature (black line). The phase change induced by the USAF target is determined by ss-QPGM to be ∼0.52 radians, whereas the calculated delay using $\frac{2\pi }{\lambda }(n_{\text{target}}-n_{\text{air}})$ is 0.52 rad, where the refractive index of the target is $n_{\text{target}}=1.52$ and the thickness, d=100  nm. Figure 2(d) presents calculated thicknesses of the phase target with seven different thicknesses, with the correlation coefficient of a linear regression determined to be ∼0.99. This agreement proves the accuracy of both the ss-QPGM measurements and the associated phase retrieval algorithm.

Next, we sought to determine the spatial fidelity of our measurements. We measured contrast (defined as the difference between the bars and slide with no sample) and plotted this as a function of line pair distance in Fig. 2(e). We note that 912.3  lp/mm (group 9, element 6) can be resolved at ∼20% contrast, which can be used as a nominal figure of merit for resolving power, and the largest spacing that results in approximately zero contrast is 1149.4  lp/mm (group 10, element 1). We also analyzed the information-theoretic bounds on resolution based on a generalized expression for spectral-spatial contrast.^32^ Since the phase is relative, the contrast forms the “signal” of the measurement, and noise can be calculated using the variance of the pixels of a large feature (here, group 6, element 1) that are away from the edges to avoid the effects of scattering. The signal-to-noise ratio (SNR) is calculated to be 24.66 for 350 nm thickness (and 11.93 for a 50 nm thickness) of the target bar, calculated by dividing the mean phase value of the region in the middle of group 6, element 1 by the standard deviation of that region. From an information theoretic perspective, the limiting resolution for this single-frequency illumination is $\frac{\lambda }{\mathrm{NA}}\frac{1}{\log_{2}\sqrt{1+\mathrm{SNR}}}$ or $\frac{\lambda }{k\cdot \mathrm{NA}}$  , where k ranges from 3 to 4. The expected resolving power of the reconstructed image is ∼300  nm, which is suitable for the chosen optics and consistent with both physical and informational limits. We note that the ss-QPGM is capable of measuring a relatively large field of view (∼345  μm×345  μm) at 20× magnification, providing opportunities to examine a statistically large number of cells (∼10  μm).

### 3.2 Live-Cell Imaging

**Fig. 3:**
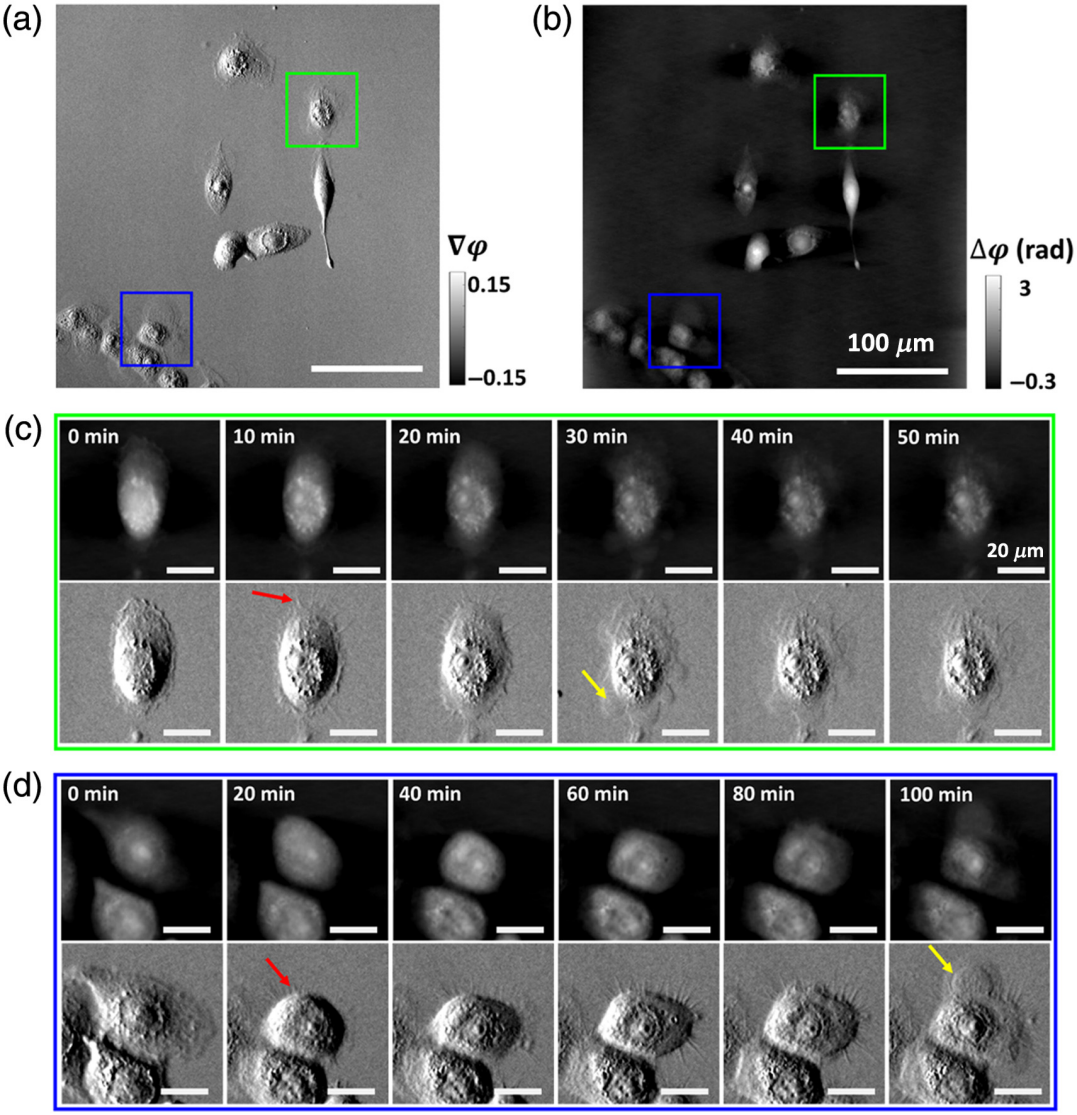
Representative ss-QPGM image frames from the time-lapse live-cell imaging data obtained for the M2 cells. (a) The gradient phase and (b) the reconstructed phase image. (c), (d) Multiple image frames [from cells indicated by green and blue boxes in panels (a) and (b)] captured at different time points during nutrient deprivation-induced cell death. Several morphological changes can be seen in different frames (see Sec. 3). Spine-like features and membrane rupture are indicated by red and yellow arrows, respectively.

We integrated an incubator with the ss-QPGM for live-cell imaging, allowing us to obtain dynamic data over the entire field of view. One application of ss-QPGM is monitoring the health of growing cells and their response to stimuli. Hence, we aimed to investigate the dynamic changes associated with cell death as observed by ss-QPGM. To explore intracellular dynamics during cell death, we conducted time-lapse imaging of living M2 cells under nutrient deprivation (see Sec. 2.3). Representative QPI images collected at different time points are shown in Fig. 3. While the overall cellular morphology was well captured, several morphological changes culminating in cell membrane rupture were also observable in both gradient phase and reconstructed phase images. Compared with cells at the initial time point (t=0), there was an increase in the number of intracellular granules, reminiscent of lipid droplets (LDs), at later stages. Spine-like features (red arrows) began to emerge within ∼10  min. These features gradually became more prominent and were followed by the formation of membrane blebs leading to eventual cell membrane rupture (yellow arrows).

### 3.3 Label-Free Cell Viability Assessment and Correlation with Live/Dead Label Assay

**Fig. 4:**
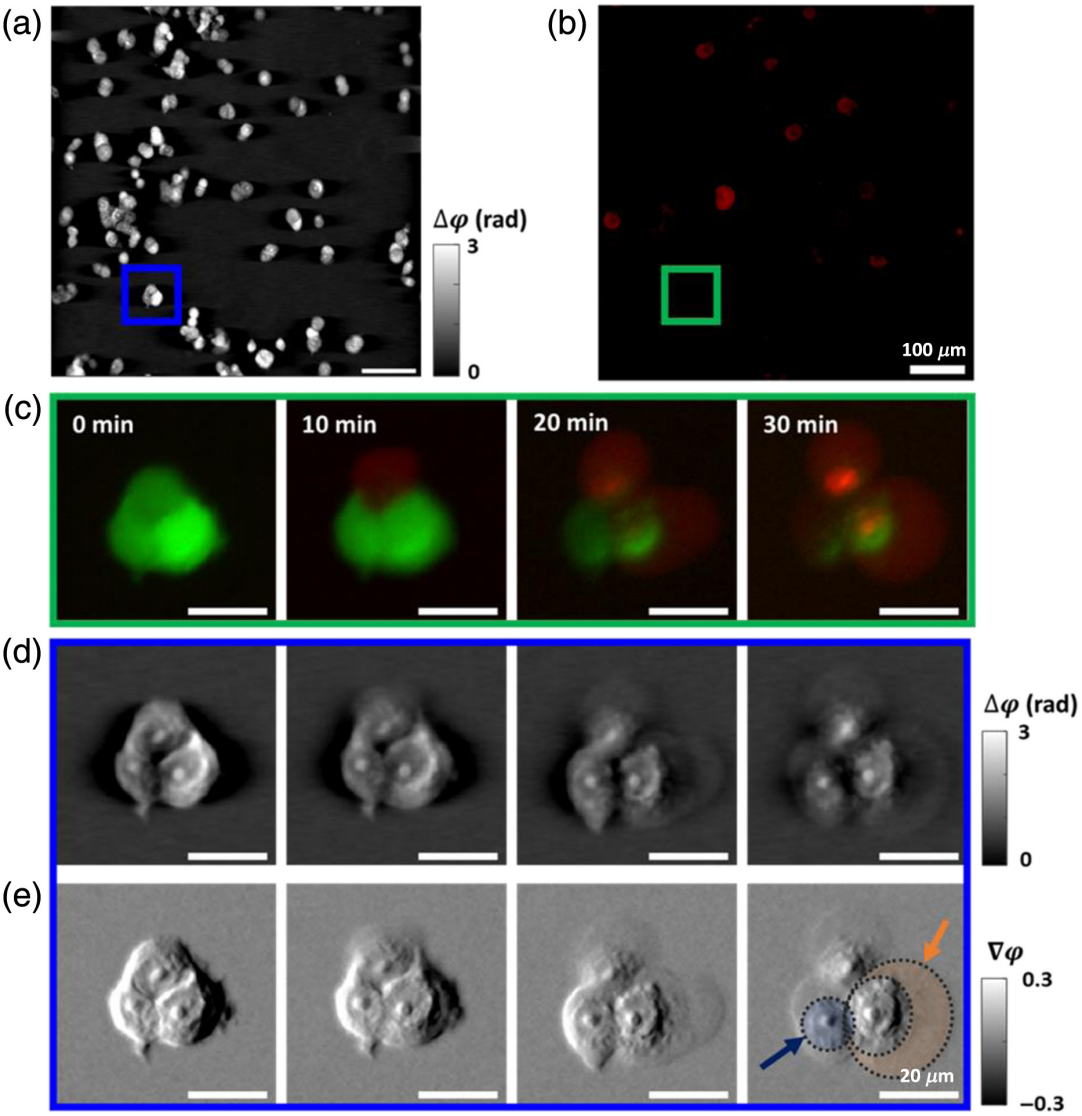
Results of in-line measurements for ss-QPGM and fluorescence during cell death induced in MCF7 by the anticancer drug ErSO. (a) The phase image and (b) the corresponding fluorescence image. (c)–(e) Multiple image frames [from cells indicated by green and blue boxes in panels (a) and (b)] captured at different time points during ErSO-induced cell death: (c) the fluorescence image. The fluorescence signal shown in green represents living cells, and that shown in red indicates progressively dying cells, (d) the reconstructed phase, and (e) the gradient phase images. Blue and orange shaded areas highlight the increased granular features and the rupture of the cellular membrane, respectively.

A fundamental sensing need in cell culture, especially for anti-cancer compound screening, is to rapidly determine the fraction of cells that are alive upon treatment with a compound. We conducted ss-QPGM imaging of ErSO-treated MCF7 breast cancer cells to examine intracellular changes induced by the biochemical cascades triggered by the drug. Since our designed system enables in-line measurements with motorized rotators in the microscope, we alternately captured fluorescence images on the same field of view, co-registered with ss-QPGM images. Here, we labeled cells for a viability assay, where living cells emit a green fluorescence signal and dead cells emit a red fluorescence signal. During live-cell ss-QPGM imaging, image frames were acquired once every minute for 3 h [Fig. 4(a)], and the corresponding fluorescent images were captured once every 10 min [Fig. 4(b)] to minimize the risk of photodamage and bleaching. Correlated fluorescence [Fig. 4(c)], reconstructed phase [Fig. 4(d)], and gradient phase [Fig. 4(e)] images collected at t=0, 10, 20, and 30 mins are shown in Fig. 4. Prominent morphological changes such as cell membrane blebbing [orange shaded area; Fig. 4(e)] and enhanced granular features (blue shaded area) were observed in the gradient phase images. Furthermore, the appearance of these morphological changes correlated with the increase in red fluorescence intensity, which indicates progressive cell death. Using the wide field of view and time-lapse imaging capabilities, specific assays can be developed for cell types using machine learning. Our goal here was not to explore the detailed chemical and morphological changes that accompany drug treatment but to show that this is possible with ss-QPGM.

## 4 Discussion

We have demonstrated real-time QPI imaging with an ss-QPGM system built on a commercial DIC microscope, utilizing spatially shifted and orthogonally polarized light transmitted through the sample.^33^ The four polarization components, separated from a single intensity acquisition and reconstructed to provide a phase image,^24^ were extensively validated using a phase-calibrated USAF target. A novelty of our study is the reconstruction method that is considerably faster than previously reported with similar hardware. While this faster approach can be applied in real time, we have also conducted a modulation transfer function (MTF) analysis to ensure that this method does indeed capture cellular shape and texture in the regime of a few cell-subcellular changes. Thus, the method reported here is appropriate for measuring dynamic changes in cells. We suggest that a similar analysis should be conducted for other reconstruction methods before they are applied to image samples such as tissues or materials that may have relatively lower-frequency spatial textures. Subtle changes in subcellular features and progressive loss of cell membrane integrity (blebbing and rupture) were revealed in the study owing to the high phase sensitivity of this method. Apoptosis and necrosis are the two major forms of cell death under physiological and pathological conditions.^34^ Apoptosis is typically characterized by cell shrinkage, nuclear fragmentation, and DNA damage, while necrosis often involves organelle damage, cell swelling, plasma membrane rupture, and the release of cellular contents.^35^ Furthermore, it was demonstrated that during nutrient deprivation, cells activate the β-oxidation of fatty acids, leading to the formation of LDs, a response similar to various other stressors.^36^ A potential link between LD accumulation and apoptosis has also been suggested.^37^^,^^38^ Our data show an increased intracellular granularity when the cells transitioned to a dead state, possibly indicating enhanced biogenesis of LDs. Other morphological changes, such as membrane blebbing and rupture, were also observed in both cell lines (Figs. 3 and 4), which are suggestive of necrosis. However, further studies are required to confirm this conjecture. Nevertheless, in the context of the anticancer drug ErSO, earlier research studies indicated that it induces necrosis in estrogen receptor α-positive breast cancer cells.^39^ Moreover, the co-registered fluorescence images show a rise in red fluorescence signal at the time of membrane rupture, which is a biochemical marker for cell death [Fig. 4(c)].

The ss-QPGM leverages intensity variations derived from the interference signals from spatially shifted beams through a sample using a DIC microscope. While ss-QGPM represents a significant advancement in high-throughput, real-time QPI of live cells, it also exhibits missing information due to an anti-symmetric response with zeros at spatial frequencies along the axis of asymmetry.^40^ This non-axisymmetric response is reflected by the phase errors and non-isotropic contrast in the resultant phase images along x- and y-directions [Fig. 2(c)]. Specifically, zero responses at the axis of asymmetry result in decreasing contrast at low-frequency features [Fig. 2(d)]. Anisotropy and non-uniform contrast of response can be seen on the square feature of the USAF1951 phase target [Fig. 2(a)], presenting a challenge in the measurement of large features or domains of a single type. The ss-QPGM technique, however, exhibits high-contrast imaging capabilities for features smaller than ∼8  μm (belonging to group 6, element 1). This size scale closely matched the size of features in the cells utilized in this study and their textures. While the concept of image quality and its impact on the resolving power of the microscope is not a concern in most implementations, we suggest that the SNR of the image could be a limiting factor, and we report this analysis. We especially advise the use of similar quantitative measures to ensure that reconstruction approaches, which may involve smoothing and its associated noise rejection, do not lead to overly optimistic assessments of the resolving power of ss-QPGM. Furthermore, in monitoring dynamic changes in a sample, phase reconstruction may not be required, and gradient measurements may be sensitively indicative of changes. However, when desired, the limitation of anisotropy responses in ss-QPGM can also be mitigated by several approaches. One method involves rotating shear direction in a DIC microscope mechanically,^29^ which allows for the recovery of isotropic phase information. However, this method introduces decreased imaging speed and potential for artifacts induced by mechanical vibration. Alternatively, modulating illumination patterns^41^ can optimize responses within the spatial frequency domain, which can suffer from low light throughput. The last strategy is employing deep learning to reduce artifacts,^42^ requiring extensive pre-acquired data sets for model training.

Further improvements in ss-QPGM imaging performance can be pursued in various directions. First, pre-experimental calibration of the misaligned polarization filter array and CMOS sensor can improve the accuracy of phase estimation. Unreliable arrangements of a polarization filter array and CMOS sensor during camera manufacturing result in inaccurate polarization pixel responses, which can account for significant phase estimation errors. The transfer function of the four polarization channels can be corrected by acquiring intensity images while rotating a linear polarizer.^43^ Second, phase estimation errors due to birefringent properties of anisotropic samples can be corrected using multiple measurements with rotating polarization components^44^ or by employing deep learning methods.^45^ The birefringence properties of specimens generate phase retardation among the orthogonally polarized states of light, potentially increasing errors in phase estimation. Sequential imaging by rotating the polarization components in a DIC microscope enables the retrieval of polarization-dependent phase images, revealing the birefringent characteristics of samples. Furthermore, the use of a deep learning approach can be considered to transform phase images recovered by ss-QPGM into images that are equivalent to those acquired by a polarized light microscope. These strategies enable ss-QPGM to generate high-contrast birefringence images of optically anisotropic structures within samples, such as amyloid,^46^ and these images can be used to demarcate tumor margins.^47^ Last, molecular-specific phase information can be integrated into this measurement using multimodal techniques. Although our ss-QGPM features an imaging speed of up to 75 Hz, the limited molecular specificity may restrict detailed cellular chemical and morphological studies. In this study, fluorescent imaging modality has been utilized to produce biochemical changes as a complement for a lack of chemical selectivity, but other modalities may also be integrated.

## 5 Conclusions

This study characterizes the performance of ss-QPGM and demonstrates its use in live-cell imaging for observing complex intracellular and morphological changes during cell death. The single-frame measurement in ss-QPGM allows it to increase its acquisition speed up to 75  frames/s, which is limited by the speed of the camera utilized. Notably, our data processing scheme bypasses the need for time-consuming and computationally intensive iterative phase recovery algorithms while yielding high-fidelity data. We demonstrate the effectiveness of ss-QPGM in examining critical intracellular changes during cell death, which revealed cell morphology dynamics leading to eventual cell membrane rupture and cell death, with exceptional clarity. We extended the capabilities of the ss-QPGM by integrating fluorescence microscopy as this multi-modal approach could fully map the morphological and biochemical facets in cell imaging studies. The development of high temporal resolution ss-QPGM, with real-time phase reconstruction and multi-modal imaging capability, holds extensive promise for sensitively studying dynamic processes in living cells.

## Data Availability

All the code, data, and materials are available from the corresponding author upon reasonable request.
